# Bread and hummus: trait connectance and correlation pleiades in grain crops

**DOI:** 10.1093/jxb/erae374

**Published:** 2024-09-07

**Authors:** Victor O Sadras

**Affiliations:** South Australian Research and Development Institute; School of Agriculture, Food and Wine, The University of Adelaide, College of Science and Engineering, Flinders University, Australia; INTA-CONICET, Argentina

**Keywords:** Heritability, phenology, phenotype, phenotypic plasticity, self-organization, seed weight, yield

**Phenotypic connectance is the level of linkage between traits. Correlation pleiades—correlations between some traits and, simultaneously, lack of correlations between these and other traits—have been interpreted as the partial independence between certain developmental processes, and as the outcome from the discrepancy between the drivers of the trait and the selective forces influencing its function. Here, I show the frequency distribution of phenotypic connectance conformed to a power law for wheat and chickpea datasets, consistent with developmental self-organization. The clustering of lower connectance for traits with higher heritability suggests a relational triangle: high connectance**⇔**low heritability**⇔**high phenotypic plasticity.**

Phenotypic integration has been investigated from evolutionary, ecological, developmental, and breeding perspectives using diverse quantification methods (Berg, 1960; Schlichting, 1989; Amzallag, 1999a, b, 2004; Armbruster *et al.*, 1999; Callahan and Waller, 2000; Pigliucci, 2003; Gianoli and Palacio-López, 2009; Damián *et al.*, 2018, 2020; Parsons *et al.*, 2020; Matesanz *et al.*, 2021; Leslie and Mander, 2023; Climent *et al.*, 2024). With few exceptions, phenotypic integration in grain crops, the focus of this article, has been overlooked.

From a developmental perspective, connectance has been defined as the level of linkage between traits (Amzallag, 2000). Development is not continuous but alternates stabilization during discrete stages (i.e. phenophases) and dismantling of relational networks between organs in the intervening, shorter critical periods (Amzallag, 2004). In this context, the critical period is a transient phase of isolation of the system that enables its evolution towards equilibrium; the transition from a dissipative to an isolated system is the source of newly emerging dissipative structures, namely new phenophases, in which environmental or developmental disturbances are adaptively integrated (Amzallag, 2004). Consistent with these propositions, integration of foliar traits in *Turnera velutina* increased from juvenile plants, which featured two functional modules related to plant defence and leaf economy, to reproductive plants with greater interconnectivity and hence lower modularity. The ontogenetic changes in foliar trait integration allowed plants to accommodate changing selective dynamics and physiological priorities through development (Damián *et al.*, 2018).

From an eco-physiological perspective, correlations are expected between traits involved in resource acquisition, defence, and stress tolerance due to resource trade-offs, multifunctionality of traits, and shared regulatory processes (Schlichting, 1989). Two examples in this context. Damián *et al.* (2020) estimated intraspecific variation in leaf functional traits related to the primary metabolism and anti-herbivore defence in *T. velutina*. Phenotypic integration of leaf traits varied 10-fold among 13 maternal families and correlated with relative growth rate and production of flowers, but not with production of seed. Amzallag (1999a) showed that priming with NaCl during early vegetative development increased tolerance to salinity in sorghum. The phase of competence for induction of this response coincides with the emergence of the first adventitious roots and was higher in genotypes with a weaker link between the seminal root and the shoot during the emergence of the adventitious root. This led to the conclusions that: (i) functional integration of the adventitious roots within the whole plant is adaptive in normal development; (ii) salt adaptation results from an integration of the environmental constraint (NaCl) during this developmental readjustment; and (iii) perturbations associated with the emergence of a new organ cause rapid variations in sensitivity required to open a competence window (Amzallag, 1999a). In both cases, the lens of phenotypic integration has been insightful.

Heritability and plasticity are negatively correlated (Lacaze *et al.*, 2009; Donovan *et al.*, 2011; Alvarez Prado *et al.*, 2014), and the links between phenotypic integration, plasticity, and heritability are inconclusive. With genotype as the main source of variation, trait connectance correlated negatively with heritability (Amzallag, 2000). With environment as a major source of variation, trait connectance correlated positively or negatively with phenotypic plasticity (Gianoli and Palacio-López, 2009; Matesanz *et al.*, 2021).

Correlation pleiades, namely correlations between some traits and, simultaneously, lack of correlations between these and other traits, have been interpreted as the independence of certain developmental processes with respect to other processes within the organism, and as the outcome from the ‘discrepancy between the agencies participating in the formation of the character and the selective forces determining its function’ (Berg, 1960). The patterns of integration among floral traits and between floral and vegetative traits initially explained in terms of pollination ecology (Berg, 1960) were later found to be species specific (Armbruster *et al.*, 1999).

Here I analyse data from field-grown wheat and chickpea to test the hypotheses that (i) phenotypic integration varies with both trait and genotype, and (ii) correlation pleiades align with trait heritability; that is, traits with high heritability such as seed weight have a lower phenotypic connectance than traits with lower heritability such as seed yield.

## Data and calculations

I analysed two datasets including 17 traits measured in 13 wheat varieties grown in four environments (Sadras and Lawson, 2013) and 20 traits in 20 chickpea varieties grown in eight environments (Sadras *et al.*, 2016). Connectance was calculated in two steps (Amzallag, 2000). First, correlation coefficients *r* were *z*-transformed to account for departure from normal distribution (Equation 1), and connectance *C*(*T*_*k*_) for each trait *T* was calculated as the average of the module of *z* (Equation 2).


$$\begin{array}{l} z=0.5\times ln\left[\left(1+r\right)/\left(1-r\right)\right]\; \end{array}$$ (1)

$$\begin{array}{l} C\left(T_{k}\right)=\left[1/\left(m-1\right)\right]\times \underset{i=1}{\overset{m-1}{\sum }}\;\left|z\right|\left(T_{k},T_{i}\right) \end{array}$$ (2)

No attempt was made to correct connectance for size (Torices and Muñoz‐Pajares, 2015). Correlation pleiades were established with cluster analysis of connectance using the Ward method standardized by column.

## The frequency distribution of connectance conformed to a power law in wheat and chickpea datasets

**Fig. 1.**
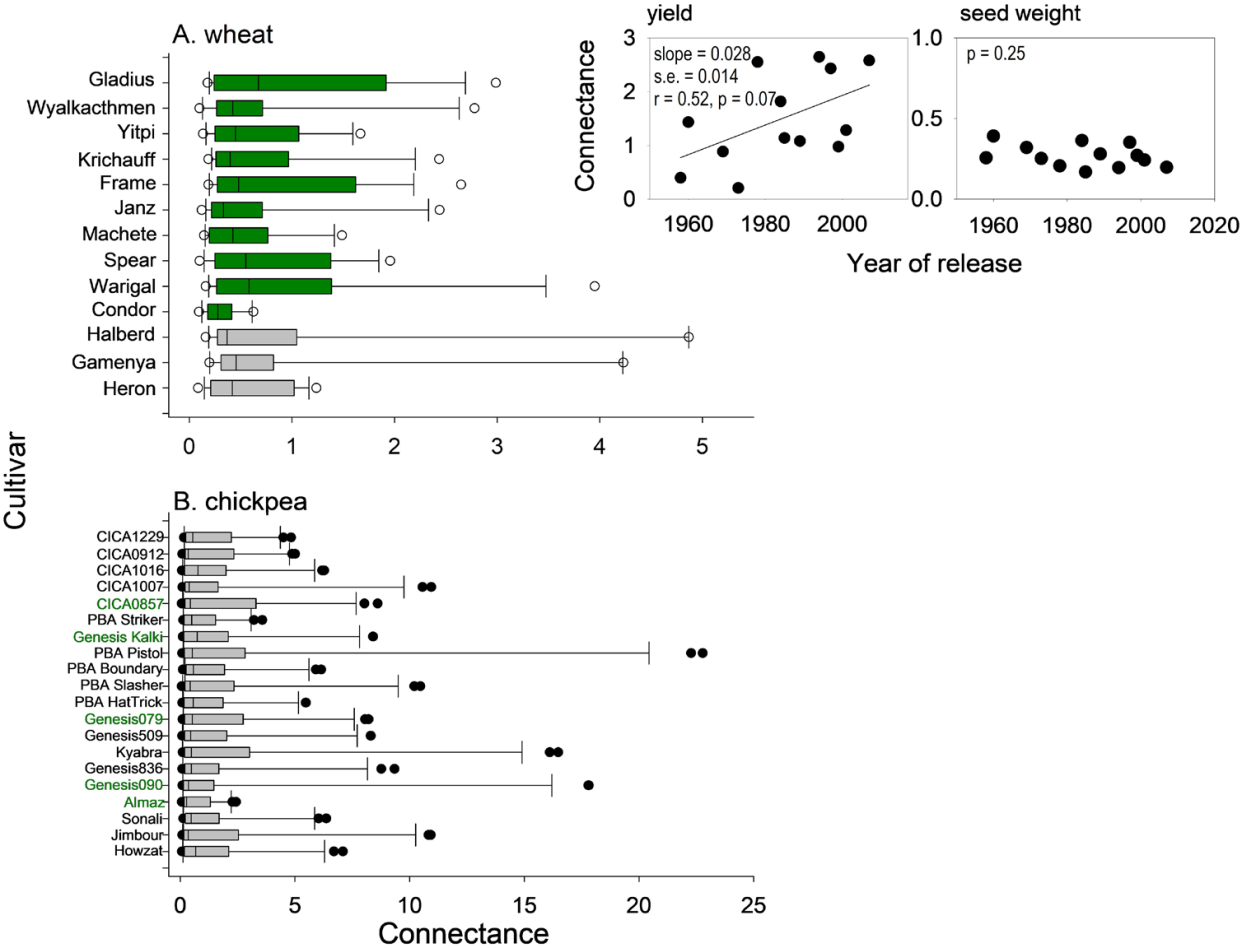
Genotype-dependent connectance in wheat and chickpea datasets. (A) Wheat, 17 traits measured in four environments. (B) Chickpea, 20 traits measured in eight environments. The scatter in connectance reflects variation among traits. Genotypes are ordered by year of release, from 1958 (bottom) to 2007 (top) in wheat, and from 2001 (bottom) to 2017 (top) in chickpea. Inset is the variation in connectance of wheat yield and seed weight with year of cultivar release between 1958 and 2007. For wheat, grey is tall, pre-green revolution cultivars, and green is semi-dwarf. For chickpea, green labels are Kabuli and black labels are Desi.

Connectance varied from 0.09 to 4.2 in wheat and from 0.06 to 22.8 in chickpea. In both crops, the frequency distribution of connectance conformed to a power law with slope of –1.665 ± 0.222 for wheat and –1.555 ± 0.126 for chickpea (Box 1A, B). Genotype-dependent median connectance in wheat varied from 0.28 in Condor to 0.67 in Gladius (Fig. 1A). In this collection of genotypes, Condor was the first semi-dwarf cultivar and Gladius was the newest cultivar (Fig. 1A). The introduction of *Rht* genes was associated with a dramatic reduction in connectance for Condor, with further breeding restoring connectance (Fig. 1A). Connectance of yield increased at 0.028 year^–1^ between 1958 and 2007 (inset Fig. 1A). In contrast, the connectance of seed weight did not vary between old and new genotypes (inset Fig. 1A); this is relevant for the relationship between connectance and heritability discussed below. Genotype-dependent median connectance in chickpea varied from 0.29 to 0.77 (Fig. 1B). In comparison with the 2.4-fold variation in wheat and 2.7-fold variation in chickpea, intraspecific variation in phenotypic integration varied 10-fold in a population of *T. velutina* (Damián *et al.*, 2020).

Box 1. **Trait connectance and correlation pleiades in wheat and chickpea**

(A, B) Frequency distributions of connectance for 17 traits measured in 13 wheat genotypes and 20 traits measured in 20 chickpea genotypes conformed to power laws. Diverse systems, including genetic and metabolic networks, fluctuations of gene expression, and size of transcriptional condensates, feature complex topology with a common property: the vertex connectivities follow a scale-free power law distribution (Kauffman, 1995; Barabási and Albert, 1999; Nacher and Ochiai, 2008; Meng *et al.*, 2024). This feature is a consequence of two generic mechanisms: (i) networks expand continuously by the addition of new vertices, and (ii) new vertices attach preferentially to sites that are already well connected. A model based on these two mechanisms reproduced scale-free distributions, which indicates that the development of large networks is governed by robust self-organizing phenomena that go beyond the particulars of the individual systems (Kauffman, 1995; Barabási and Albert, 1999). Power law distributions are consistent with, but do not prove, self-organization. With a focus on connectance, Amzallag (1999b) advanced a biological explanation for a role for self-organization in developmental processes. Multiple contemporary perspectives converge to support a significant role for self-organization in biological development (Heylighen, 2023; Kauffman and Roli, 2023; Baverstock, 2024).

(C, D) Hierarchical clustering of trait connectance partially aligns with heritability. For both wheat and chickpea, connectance was low for seed weight in alignment with the high heritability of this trait, usually above 0.7 (Harper *et al.*, 1970; Sadras, 2007). For wheat, time to flowering had the lowest connectance of all 17 traits tested, in correspondence with the high heritability of this trait, for example broad sense heritability of time to heading and flowering between 0.7 and 0.9 (Dunckel *et al.*, 2017). For chickpea, phenological development had high connectance, above 2; this compares with lower heritabilities, for example <0.5 for time to flowering and <0.4 for podding from parent–offspring correlations (Anbessa *et al.*, 2006). Whereas daylength and temperature are drivers of phenological development in both wheat and chickpea, soil factors such as moisture and salinity also modulate phenological development of pulses (Lizarazo *et al.*, 2017; Li *et al.*, 2022); a larger environmental component of the phenotypic variance can therefore account for the lower heritability of phenological development in chickpea and its associated higher connectance. In both crops, connectance of yield was at the higher end of the range in correspondence with the lower heritability of yield (Cooper *et al.*, 1995; Yang *et al.*, 2005; Hoffmann *et al.*, 2009).

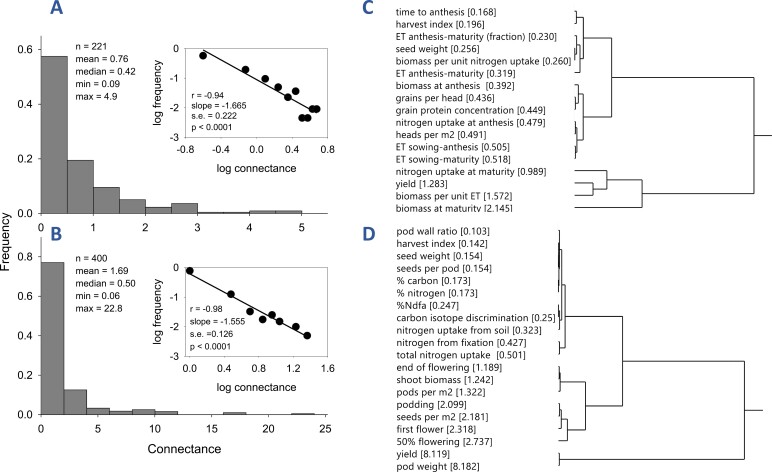

A negative relationship between connectance and heritability calculated from parent–offspring relationships in sorghum has been interpreted in two alternative ways: (i) pre-existing information, as quantified with heritability, is the single source of developmental control whereas the linkage between traits generates a ‘developmental noise’ that disturbs the expression of this pre-existing information or (ii) the network of relationships is also a source of information for development, ‘which completes, substitutes or even counteracts expression of pre-existing information’ (Amzallag, 2000). Contradictory evidence, which supported both (i) and (ii), was interpreted with a speculative model based on the variability of connectance whereby (i) applies above and (ii) applies below a threshold of connectance: ‘below a critical value of lability, expression of a character is controlled by its connectance… and… an increased lability in connectance reduced its involvement in character expression, which became probably mainly determined by a pre-existing information’ (Amzallag, 1999b). Further considerations on cell-to-cell signalling during development led to the conclusion that ‘the metacellular network is not directly determined by pre-existing information but is an expression of the self-organization dimension of development. Thus, although related to genetic expression, connectance may be considered as an autonomous dimension in development’. The conclusion that morphogenesis involves both genetic and self-organizing dimensions has implications for the reliability, stability, and adaptability to the developmental processes (Amzallag, 1999b). From a teleonomic perspective, Levin (2023) also concludes that ‘development is incredibly reliable, producing bodies to very tight tolerances despite considerable deviations and noise at the level of gene expression and cellular activity’ … ‘development is not hardwired but context-sensitive and plastic’.

The emphasis on ‘datasets’ in the title of this section is to caution against generalizations; the findings reported here are specific for the combinations of crop species, traits, and growing conditions; other datasets may feature different frequency distributions, for example log-normal (Broido and Clauset, 2019). A small sample from an experiment comprising eight traits measured in six sorghum lines grown in a single environment (*n*=48) returned a close-to-normal frequency distribution of connectance (Shapiro–Wilk test *P*=0.06), with a range from 0.17 to 1.14, median=0.63, and mean=0.60 (calculated from data in Amzallag, 1999b).

## Correlation pleiades were apparent for both wheat and chickpea datasets

Correlations between traits are commonplace in crop science. Positive correlations do not prove, but are a first step towards, demonstration of causal relationships (e.g. Calderini *et al.*, 2021). Negative correlations are mostly interpreted in terms of trade-offs (e.g. Du *et al.*, 2020). Beyond these perspectives, interpretation of correlations would benefit from alternative theoretical contexts.

The influential notion of Berg (1960) of correlation pleiades allows for developmental and evolutionary interpretations of plant phenotypes. From this lens, Berg (1960) concludes that ‘correlation coefficients between the dimensions of grains and those of other parts of the organisms are somewhat smaller than all other correlation coefficients. This might well be expected, the grain being essentially an organism of the next generation with a genotype of its own, and not a part of the parent organism’. The typically high heritability of grain weight and grain dimensions has been interpreted in terms of the mother–offspring conflict of Smith and Fretwell (1974) (Sadras, 2007). The low connectance of seed weight in both wheat and chickpea (Fig. 1C, D), and the negative association between heritability and phenotypic plasticity (Lacaze *et al.*, 2009; Donovan *et al.*, 2011; Alvarez Prado *et al.*, 2014) completes a relational triangle: high connectance⇔low heritability⇔high phenotypic plasticity.

## Conclusion

Phenotypic integration has received attention in ecology and evolution but is not part of our current thinking in crop sciences. We propose that studying the genotype- and trait-dependent variation in phenotypic integration could be useful to understand and manipulate plant development against the relational triangle with connectance, heritability, and plasticity vertices. A testable prediction from this perspective is, for example, that the slowing down of genetic gain in the yield of wheat, attributed to diminishing returns and harvest index reaching its biological limit, is also the consequence of diminishing heritability associated with higher yield connectance.
